# Rehabilitation interventions at senior day centres for older adults: a scoping review

**DOI:** 10.3389/fpubh.2025.1659493

**Published:** 2025-11-20

**Authors:** Marie Jönsson, Cecilia Pettersson, Mialinn Arvidsson Lindvall

**Affiliations:** 1 Department of Health Sciences, Örebro University, Örebro, Sweden; 2 Department of Health Sciences, Hogskolan Kristianstad, Kristianstad, Sweden; 3 Department of Health Sciences, Malardalens Universitet, Västerås, Sweden

**Keywords:** day care, interventions, elderly, professions, tools

## Abstract

**Background:**

There is ample evidence that senior day centres can provide rehabilitation, increase activity and support social participation for older adults.

**Aim:**

The aim of this scoping review is to provide an overview of the scientific literature in this field and investigate whether there is scientific evidence for the efficacy of rehabilitation interventions at senior day centres for older adults.

**Methods:**

The scoping review was performed in accordance with the Preferred Reporting Items for Systematic Reviews and Meta-Analyses extension for Scoping Reviews (PRISMA-ScR). Data were collected from databases between January 2010 and December 2020 and updated December 2023. The scoping review was conducted in the databases CINAHL, Cochrane Library and PubMed and 29 articles were identified for inclusion.

**Results:**

Rehabilitation interventions at the senior day centres varied considerably. The main findings concern interventions of a physical, cognitive and/or social character. The most common dimensions of outcome were improvements in physical and cognitive ability and quality of life. There was also considerable variation in the professionals involved. Other important factors to observe when participating in interventions at senior day centres could be loneliness and risk of falls.

**Conclusion:**

While rehabilitation interventions at senior day centres are striving towards person-centred care, there is room for improvement to meet older adults’ individual needs. In conclusion, this scoping review shows that greater focus is needed to strengthen the evidence base for examine interventions that are most effective in these settings.

## Introduction

Senior day centres provide rehabilitation in community-based services that provide support and create opportunities for older adults to engage in various activities and interact with their peers (1). Senior day centres are characterised as facilitators of activity, companionship and social interaction (2–4), fostering independence and enabling older adults to age in place while minimising avoidable hospitalisations (5). These centres also offer services intended to promote active and healthy ageing (2), although it is notable that they are described in terms of various interventions.

In healthcare and social care for older adults, senior day centres are described as preventing ill health and loneliness (1, 2, 6). For example, these centres act as a point of delivery of services for an estimated 46 million older adults in the United States (7) and can provide activities that promote social interactions and healthy ageing (2, 6). In this study, the term health outcomes refer broadly to physical, cognitive, and social dimensions of health among older adults, which is in line with the World Health Organisation’s holistic definition of health (8, 9). These outcomes may include improvements in mobility, cognitive functioning, emotional well-being, social participation, and perceived quality of life (8, 9). Senior day centres are described as community-based services including rehabilitation interventions designed to support older adults living at home by providing structured daytime programmes that combine social, and physical activities. These daytime programmes include opportunities for social interaction and engagement in meaningful activities, access to rehabilitation and preventive care provided by various professions and may also give respite support for informal caregivers (10, 11). In order to support healthy ageing, the World Health Organisation (WHO) has developed a policy framework that defines healthy ageing as “*the process of developing and maintaining the functional ability that enables wellbeing in older age*” (9). Functional ability is also dependent on interactions with relevant environmental characteristics. These environmental characteristics include factors such as policies, the built environment, relationships, values and attitudes (9). Interaction with other people is valuable; loneliness is a major risk factor for physical and mental illness, as well as frailty (12). As a confounding effect for physical and mental health in later life, loneliness and social isolation among older adults have therefore garnered significant attention in recent decades (13, 14). Maintaining social contact with other people facilitates healthier ageing (13). To this end, well-functioning municipal senior day centres are described as important for relieving the burden on assisted-living services, home-care services and primary healthcare (1, 6), where staff are often stretched to the limit. Previous research on senior day centres in general has shown that rehabilitation interventions are diverse in order to attract a wide range of clients (2), create opportunities to maintain meaningful activities, encourage social interactions (2) and reduce feelings of loneliness (2, 6, 15). It is unclear whether the rehabilitation interventions offered at senior day centres meet the direct needs of the older adults. For example, whether the types of physical and social activities offered are person-centred and address older adults’ interests and individual needs. To enable person-centred care at senior day centres, it is important to investigate whether these interventions are individualised and needs-based. According to a person-centred approach, both clients and staff must be involved in planning interventions (16).

With regard to existing research into senior day centres for older adults, we conclude that the empirical evidence is limited. In a broader healthcare context, senior day care is a service with the potential to help older adults remain healthier and support them to age in place. Furthermore, attending a senior care centre can help to maintain abilities that can potentially prevent unnecessary hospitalisation or institutional care (5, 17). An understanding of interventions, outcomes, tools and the involved professional practitioners is vital to developing future complex, multicomponent interventions (18). In order to broaden knowledge concerning the subject, a scoping review was conducted (19). The aim of this scoping review was to summarise the range of rehabilitation interventions implemented in senior day centres for older adults in terms of outcomes, tools and professional involvement.

## Method

A scoping review approach was chosen to gather and summarise literature and identify knowledge gaps (20). To address the limited research available, this scoping review provides an overview of existing studies and highlights emerging evidence on the topic (20). Using the Preferred Reporting Items for Systematic Reviews and Meta-Analyses extension for Scoping Reviews (PRISMA-ScR) (21), we undertook a systematic literature search of articles published in international journals. The following stages were implemented: (1) identifying the research question; (2) identifying relevant articles; (3) selecting articles; (4) charting the data; (5) collating, summarising and reporting the results; and (6) consulting. In reporting, we adhere to the PRISMA guidelines for scoping reviews (21). The literature search was conducted in spring 2020 by an academic librarian together with the last author and was subsequently updated by the first and last author in December 2023. All authors have substantial experience in rehabilitation of older adults and rehabilitation interventions. The first and second authors are occupational therapists (OTs), and the last author is a physiotherapist (PT).

**Figure 1:**
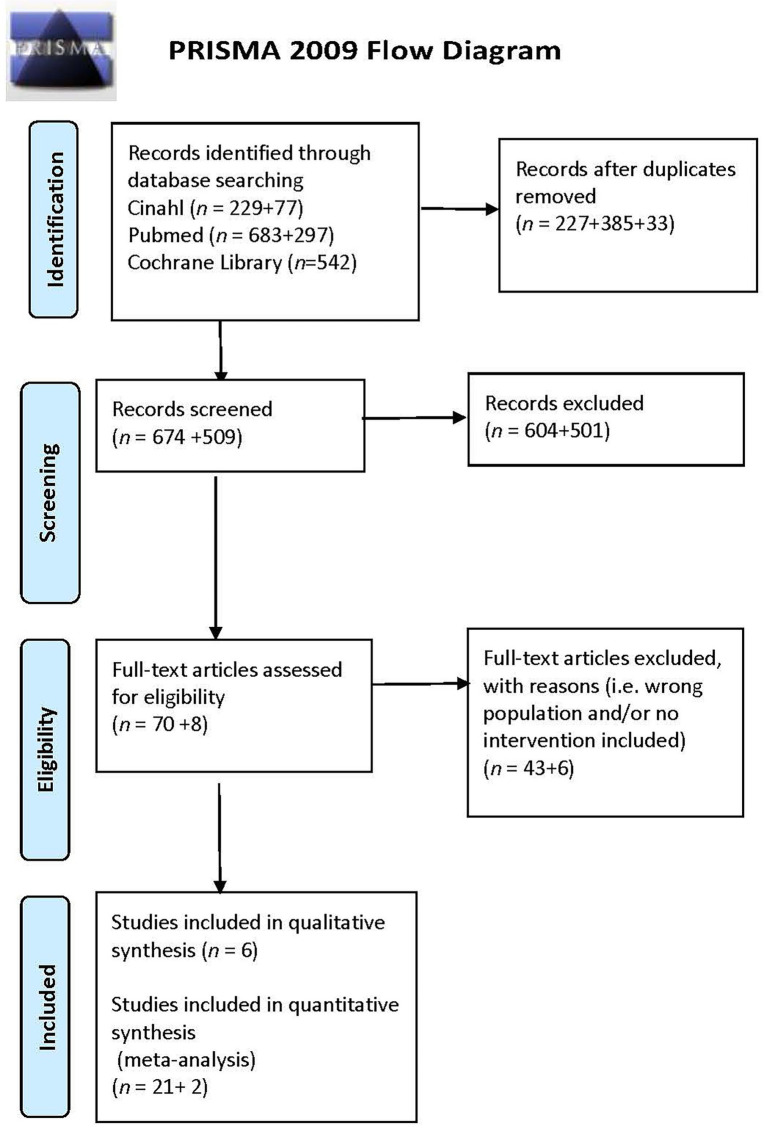
PRISMA flow diagram from Moher et al., 2009.

1. **Identifying the research question**. Based on the aim of the scoping review, the following research questions were formulated:

- What interventions were implemented?
- Which outcome dimensions were used to capture the effects of those interventions?
- What tools and assessment methods were used?
- What effects of the interventions have been demonstrated?
- Which professions have been involved in the interventions?

2. **Identifying relevant articles**. A systematic literature search was conducted in the databases CINAHL and PubMed, using the following keywords in various combinations: Senior Centres, Adult Day Care Centres, Day Care, Medical, Day care program, physical and creative. The selection criterion was studies published in international peer-reviewed scientific journals between January 2010 and December 2020 (later updated in January 2021 and December 2023) involving subjects 65 years of age or over who attend senior day centres. An additional search was conducted in Cochrane Library for the same search period. Studies published in a language other than English were not considered. Since different databases use different concepts for the same phenomenon, each specific keyword was modified for each database and the search was adapted for the different databases. The limit “aged 65 + years” was applied to the literature search. All types of studies were considered. No grey literature was considered for inclusion in this scoping review. Grey literature was excluded because it was deemed unlikely to provide evidence relevant to the research questions concerning senior day centres (22).
3. **Selecting the articles**. As recommended by Tricco et al. (21), the selection process is presented as a flow diagram (Figure 1). The first and last authors reviewed the articles independently and examined the titles and abstracts of the articles identified. The Rayyan systematic review programme was used by the authors when performing their independently systematic work (23). The first step was to assess whether the abstracts were potentially relevant for inclusion. In the second step, the first and the last author independently screened the records, resulted in 29 full-text studies. Thereafter, all three authors independently read these studies abstracts and then discussed the studies in relation to inclusion and exclusion criteria. The same three authors subsequently read the remaining full-text studies in full text to reach a consensus on which original publications met the selection criterion. All publications included in this review are presented in Table 1.
4. **Charting the data**. According to the research questions formulated for this study, data were synthesised systematically following the reporting guidelines provided by PRISMA-ScR (21) and Arksey and O’Malley’s methodological framework (20). After initially conducting independent screenings in the Rayyan systematic review software, the first and last authors discussed any conflicting inclusion decisions until consensus was reached. Following this, data extraction was carried out using a structured data charting process, designed to present the information in a clear and logical manner for the reader (24). The extracted data included, for example, author(s), year of publication, country of origin, study aim, population, methodology, and findings relevant to the research questions. Results from the selected studies (*n* = 27) were then systematically extracted and transcribed in relation to the predefined research questions. This process was applied consistently across all included studies (Table 1). Several joint discussions took place between all authors in the research team regarding the relevance of articles in relation to the inclusion criterion.
5. **Collating, summarising and reporting**, is described in the Result section.
6. **Consulting**, optional and was not included in this scoping review.

**Table 1:** Characteristics of the included articles.

Author(s)/year of publication/Country of origin /Title	Study aim	Study design/methods	Study population/sample size	Outcome measure	Summary findings
Armadans et al. (38), Spain 2015 Empowering Senior Citizens in Leisure Settings Through Mediation: Becoming a Mediator	To explain the implementation of a programme and its results, a programme that seeks to develop the “senior citizens” figure and to empower senior users of leisure facilities with basic social skills.	Quantitative Explorative *(Questionnaire)*	A self-assessment questionnaire was applied with 32 adults (10 male and 22 female; mean age 65.46 years) to evaluate mediation skills learned before and after a workshop with 0-to-10 rating.	“Custom-made” instruments designed to establish baselines (before the intervention) and to assess the acquisition of knowledge at the end of the programme. Six-question self-assessment questionnaire was applied with 0-to-10 rating.	Participants demonstrated significant improvements in conflict understanding, communication, and conflict management skills. Empowering senior citizens with mediation training in leisure centres proved to be an effective approach to fostering active and competent ageing.
Ayalon (39), Liat, Israel 2019, Subjective Social Status as a Predictor of Loneliness: The Moderating Effect of the Type of Long-Term Care Setting	To examine the role of subjective social status as a predictor of loneliness in adult day care centres (ADCCs) and continuing care retirement communities (CCRCs) over a 1-year period.	Quantitative Comparative *(Face- to-face interviews conducted within two separated occasions, spread about 1 year apart)*	The main analyses consist of data from 245 respondents (141 ADCC participants and 104 CCRC residents), 2016 and 2018.	UCLA Loneliness Scale MacArthur Scale of Subjective Social Status	A significant interaction was observed between subjective social status and type of long-term care setting. The findings provide insights into the temporal relationship between two subjective experiences: loneliness and perceived social status.
Boen, et al (44) Norway, 2012,	The objectives were to examine the effect of a preventive senior centre group programme consisting of weekly meetings, on social support, depression and quality of life.	Qualitative A randomised controlled trial	A questionnaire was distributed to a random sample of 4,000 individuals aged 65 years and older in Oslo, resulting in 2,387 completed responses. These respondents constituted the recruitment base for the trial, with HSCL-10 scores applied as the primary inclusion criterion. In total, 138 participants were randomised to either the intervention group (*n* = 77) or the control group (*n* = 61). The final analyses were conducted on data from 92 participants.	HSCL-10, OSS-3, BDI, and measures of life satisfaction, health, and perceived benefits of the intervention.	The intervention produced modest benefits, including slightly increased social support, smaller rises in depression, and a less pronounced decline in life satisfaction compared with controls. No significant effects were found for self-reported health, yet participants appreciated the programme and increased their engagement with the centre.
Brataas, et al., (25) Norway, 2010, Experiences of day care and collaboration among people with mild dementia	To provide insight into how older adults with mild cognitive impairment perceive and experience day care.	Qualitative Descriptive, narrative hermeneutic method,	The study included nine Norwegian clients (age 77–88 year), living at home with mild dementia to assess how a day care programme once a week, for seven weeks, with group collaboration and social and cultural activities was experienced by clients, 2007–2008.	Narrative interviews	Participation in collaborative day care was associated with greater feelings of meaning, well-being, and contentment, with safe transfer identified as a prerequisite for engagement. Further research is needed on facilitating person-centred, collaborative day care across diverse cultural contexts and among younger seniors with mild dementia.
Battaglia, et al. (43) Palermo, Italy, 2016 Effects of an adapted physical activity programme on psychophysical health in elderly women	To assess the effectiveness of a specific adapted physical activity (APA) intervention programme in the improvement of the health-related quality of life (QOL) and functional condition of spine in elderly women.	Quantitative Comparative	The APA programme was conducted for 8 weeks with two training sessions/week. Thirty women were recruited from a senior centre and randomly assigned to two groups: Trained group (TG; age: 68.35 ± 6.04 years) and control group (CG; age: 69.69 ± 7.94 years). CG did not perform any physical activity during the study. 2012	Bodyweight and height. Spinal Mouse(®) (SM) The Short Form Health Survey 36 (SF-36) Mental Component Summary Score (MCS-36)	An eight-week adapted physical activity programme improved psychophysical health in older adults, highlighting the importance of maintaining an active lifestyle to enhance quality of life and physical fitness in ageing populations.
Chan et al. (26), Taiwan, 2020 Effects of an Active Music Therapy Programme on Functional Fitness in Community Older Adults	To test the effectiveness of a 3-month active group music therapy programme on the functional fitness of community older adults in Taiwan	Quantitative Quasi-experimental study *(Repeated measures)*	146 community-dwelling older adults. All of the participants were assigned either to the active music therapy intervention group (three senior centres, *n* = 77) or to the comparison group (four senior centres, *n* = 69) based on the expressed desire of each centre. Active music therapy group were performed in the experimental group twice weekly for a period of 3 months and comprised 24 sessions	Demographic information Barthel Index Short Portable Mental Status Questionnaire (SPMSQ) Cardiopulmonary fitness: 2-min step test Body flexibilities: back-scratch test and chair sit-and-reach test Muscle power and endurance: hand grip test and 60-s chair stand test: A digital handgrip dynamometer (Electronic Handgrip Type Model: TTM) Balance: open-eye single-leg stand test	Active group music therapy significantly improved multiple aspects of functional fitness—including cardio-pulmonary fitness, flexibility, muscle strength, endurance, and balance—in community-dwelling older adults, with effects sustained over 3 months.
Eaton (27), USA, 2022 Let us progress! Implementing professionally led arts-based programming in senior centres	To describe the development and evaluate the implementation of three pilot programmes (1Readers theatre, 2 Choir, 3 Improvisation/movement)	Mixed method/ Quantitative *(Open ended interviews Observations Questionnaire)*	The pilot programmes: 1 Readers theatre (9 participants, mean age 76 years) 2 Choir (13 participants, mean age 70 years) 3 Improvisation/movement (9 participants, mean age 73 years). Each programme was offered once a week for 1 h during 10 weeks. A total of 35 participants participated at least 1 week in all programmes	Demographic information Patient-reported Outcomes Measurements Information System (PROMIS) to evaluate satisfaction, emotional distress-depression, social isolation, and cognitive abilities Self-reported Health Field notes Open-ended interviews with teaching artists, senior centres directors and three participants	Overall satisfaction was high across all programmes, with participants expressing enjoyment with courses that offer a challenge and desired that courses continue. The choir had the highest average of regular attendance, while the improvisation/movement class struggled with recruitment.
Finnanger et al. (45), Norway,2020Physical activity in people with dementia attending farm-based dementia day care - a comparative actigraphy study	To investigate the potential of farm-based day care services as services that can promote physical activity for people with dementia.	Quantitative *(Comparative)*	Data from two separate studies with persons 65 years and older was used. Participants farm-based day care *n* = 29 Participants regular day care *n* = 107 Age 60-	Demographic information ActiSleep+Clinical Dementia Rating (CDR) Scale Timed up and Go –test (TUG)	Participants in farm-based day care for people with dementia were younger and engaged in higher levels of moderate physical activity than those in ordinary day care, suggesting that farm-based programmes promote greater physical activity among attendees.
Galinha et al. (28), Portugal, 2022 Sing4Health: Randomised controlled trial of the effects of a singing group programme on the subjective and social well-being of older adults	To analyse the effects of a singing group intervention on participants’ subjective and social well-being	Mixed method/ Quantitative *(RCT design and interviews)*	A 34 session singing group programme (SGP). Participants (Mean age 76.66 years; SD8.79) were randomly allocated to the intervention group (*n* = 89) and the control group (*n* = 60). Data was collected at baseline, 4 months after baseline assignment and 6 months follow-up. Data collection took place for 10 weekdays, from 10–15 participants per day.	Demographic information Satisfaction with Life Scale The Positive and Negative Affect Schedule The Scale of Social Well-Being WHOQOL WHOQOL-OLD UCLA Loneliness Scale The Four-Item Measure of Social Identification The Rosenberg Self-Esteem Scale Motivations collected with semi-structured interview.	The Social Gardening Programme (SGP) produced significant and sustained improvements in positive affect and social well-being, with marginal gains in self-esteem. Qualitative findings supported the quantitative results, and mediation analyses indicated indirect effects of social identification and self-esteem on affect and loneliness.
Ganz et al. (50), Israel, 2014 The effect of humour on elder mental and physical health	To examine the effect of a five month intervention using a humour workshop among a sample of Israeli community-dwelling elderly people who attended senior centres	Quantitative *(Questionnaires)*	The sample consisted of 92 subjects, 42 in the control group and 50 in the humour workshop group Most of the participants were females (n ¼ 67, 74.4% among those who responded) either married (n ¼ 45, 48.9%) or widowed (n ¼ 45, 48.9%). The mean age of the sample was 76.9 (SD e 6.8).	Self-administered questionnaires: RAND Health Status Questionnaire 17 shortened version *(12- item)* The General Well Being Scale (GWB) *(18-item)* The Brief Symptom Inventory (BSI) *(53-item)*	Participants in the humour therapy workshop showed reduced anxiety and depression and improved well-being compared with controls, though no effects were found for general health or quality of life. The findings suggest humour workshops may beneficially influence mental health and warrant further evaluation.
Gjernes (51), Trude; Norway 2017 Knitters in a Day Centre: The Significance of Social Participation for People With Mild to Moderate Dementia	To explore how people with dementia interact and solve problems while participating in social activities.	Qualitative Explorative *(Ethnographic approach)*	Number of participants varied in between 6–9 a day, and were observed at a day centre in a Norwegian city during a period of 8 weeks, 2014.	Data were collected with participant observations and field notes between 4 and 6 h at a day care centre.	The social activity of knitting facilitated conversations about different topics, required various forms of memory and problem solving, and involved different participant statuses. Being part of the knitting group appeared to help the participants maintain their skills and facilitated sociability
Hedayati et al. (29), Iran, 2022 Effect of Group Physical Games on Life Quality of Older Adults at Adult Daycare Centres	To investigate the effect of group physical games on life quality (QOL) of older adults at an adult day care centre.	Quantitative A quasi-experimental design (*pre- and post-cohort design*)	50 participants in the intervention and in the control group. Participants (*n* = 25) in the intervention group received physical games programme twice a week for 6 weeks.	Demographic information The SF12	The quality of life scores of the participants in the intervention group have significantly improved compared to the control group (*p* < 0.001). Physical exercise programme (especially stationary physical game) can improve quality of life in both physically and mentally aspects.
Iecovich, E. Biderman (30), Israel 2013, Attendance in adult day care centres of cognitively intact older persons: reasons for use and nonuse	To examine the reasons for nonuse of adult day care centre (ADCC), and explore the reasons for use of ADCCs among users.	Quantitative A quasi-experimental design	Face- to –face interviews were conducted with a structured questionnaire. The sample included 819 respondents of whom 417 were users of 13-day care centres and 402 are nonusers, matched by age, gender, and family physician in the southern region of Israel, 2009–2010.	Users: Instrumental activities of daily living (IADL) Activities of daily living (ADL) Self-Rated Health Economic Status Comorbidity Nonusers: A list of 20 items list of reasons for ADCC underuse from 20 items was composed and used.	Use of Adult Day Care Centres (ADCCs) enhanced well-being, met individual needs, fostered social relationships, and reduced caregiver burden. Barriers to use included accessibility, participant and centre characteristics, perceived lack of need, and personal difficulties.
Keisari et al. (31), Israel, 2020 Playback theatre in adult day centres: A creative group intervention for community-dwelling older adults	To provide an evidence- informed framework for drama therapy interventions, which would allow older adults to bring up and explore their life-stories in a dramatic creative process in their own community.	Qualitative Phenomenological perspective	A playback theatre intervention consisting of 12 sessions (90 min each) involved 27 participants (aged 63–91, mean 79.3 years; 19 females) whose creative processes were videotaped and analysed through post-intervention interviews. Additionally, 13 staff from 13 adult day centres participated in 45–60 min focus groups to explore further effects of participation.	Video recording with 2 cameras, producing 36 sessions of three groups. Three post focus groups.	The use of play back theatre seem to have the potential to serve as a creative intervention in ADC communities and expand it to enable a person’s social engagement in the community. The result imply the potential benefits of using playback theatre groups to supplement the routine care in ADCs.
Kim et al. (49), Japan 2011, Use of senior centre and the health-related quality of life in Korean older adults	To examine the relationship between the use of senior centre and health-related quality of life in Korean older adults.	Quantitative Comparative	A questionnaire survey was conducted to two 154 older adults(aged 71.2 ± 3.7 years, male 19.3%, female 80.7%) who used a senior centre, respectively, 137 older adults (aged 70.2 ± 4.8 years, male 39.4%, female 60.6%) who did not use a senior centre.	Demographic information were obtained from a questionnaire. The Korean version of short-form 36-item health survey (HRQOL) was administered to assess the health-related quality of life.	The 8-domain scales of physical function and role-physical were significantly higher in the users of the senior centre compared with the non-users.
Kogan A. C et. (46), USA 2013 Be Well: results of a nutrition, exercise, and weight management intervention among at-risk older adults	To test the effectiveness of a multifaceted exercise and nutritional education intervention for chronically ill, community-dwelling older adults.	Quantitative A pre-post cohort design	Data collected via 4-month in-person interview and telephone follow-up at two community-based senior centres in Los Angeles. Participants (*n* = 62) were aged 60 years or older, with multiple chronic conditions and one or more emergency department visits or hospital admissions in the previous 6 months, and at nutritionally moderate to high risk. October 2006 to June 2008	Baseline data were collected via in-person interviews and included self-reports of physical activity and depression, and body and fitness measurements. Follow-up data were collected through in-person assessments (body and fitness measurements) during the last Be Well session and telephone (physical activity and depression).	Participation in Be Well was associated with physical and emotional health improvements among participants. Nearly half the sample exhibited depressive symptomatology at baseline, which decreased by 64% at follow-up. This reduction in depression may be attributed to several aspects of the intervention.
Liao et al. (47), Taiwan 2020 Using virtual reality-based training to improve cognitive function, instrumental activities of daily living and neural efficiency in older adults with mild cognitive impairment	To explore the effects of VR-based physical and cognitive training on cognitive functions, brain activation, and IADL, as well as comparing the VR intervention to a traditional combined physical and cognitive training programme.	Quantitative Single-blinded randomised controlled trial. A pre-post cohort design	Older adults with mild cognitive impairment (MCI) were randomised into virtual reality (VR)-based physical and cognitive training (*n* = 18) or a combined physical and cognitive (CPC) training (*n* = 16) for 36 sessions during 12 weeks. Changes in prefrontal cortex activation were also captured	Demographic information MoCA The Executive Interview 25 (EXIT 25) The Chinese version of the Verbal Learning Test (CVVLT) The Lawton Instrumental Activities of Daily Living scale (IADL) Near-infra red spectroscopy (NIRS) measure brain activation	Both groups improved in executive function and immediate verbal memory, but only the VR group showed significant gains in global cognition, delayed verbal memory, and IADL. VR-based physical and cognitive training enhances cognitive function, daily living skills, and neural efficiency in older adults with MCI.
López-García et al. (48), Spain, 2022 Feasible Intervention through Simple Exercise for Risk of Falls in Dementia Patients: A Pilot Study	To determine the effectiveness of simple lower limb strength and single leg stance training, which would be feasible in the facilities of day care centres, in reducing the risk of falls in people with dementia and to analyse whether sex, age, and time since dementia diagnosis affect the effectiveness of the intervention.	Quantitative A pre-post cohort design	Participants (*n* = 20) were divided in intervention and control groups. Exercise sessions for 45–50 min on weekdays for 5 weeks.	Demographic information Barthel Index Tinetti Mobility Test Physical Performance Battery (SPPB)	The result showed that performing simple routines consisting of sit-to-stand and single leg stance for few minutes every weekday were able to improve functional ability after 5 weeks in older adults with dementia.
Marijeke van Haeften-van Dijk et al. (52), 2017, Netherlands, Is socially integrated community day care for people with dementia associated with higher user satisfaction and a higher job satisfaction of staff compared to nursing home-based day care?	This study examined whether community-based day care with carer support, following the effective Meeting Centres Support Programme model, is associated with greater satisfaction among people with dementia and their informal caregivers, as well as higher job satisfaction among care staff, compared to traditional nursing home-based day care.	Quantitative Pre- and post-comparison, 6 months	Data were collected in 11 NH day care centres and 11 CO day care centres. User satisfaction of PwD and CG was evaluated in the 11 NH day care centres (n_PwD_ = 41, n_CG_ = 39) and 11 CO day care centres (n_PwD_ = 28, n_CG_ = 36) with a questionnaire after 6 months of participation.	User satisfaction of persons with dementia: a 13-item study specific survey. User satisfaction of informal caregivers focused on involvement, activities and support. Job satisfaction among staff: Leiden Quality of Work Questionnaire (LQWQ) Work experience among staff: Questionnaire Experience and Evaluation of Work (QEEW)	User satisfaction of persons with dementia: a significantly higher satisfaction in users of community-based day care. The users rated the activities more positively. Informal carers: moderately significant differences between groups in favour of CO day care. Job satisfaction: significant moderate improvement (*r* = 0.54) in satisfaction with work pace in community-based day care.
Murphy et al. (32), 2017, UK, Health benefits for health and social care clients attending an Integrated Health and Social Care day unit (IHSCDU): a before-and-after pilot study with a comparator group	To identify whether attendance at an integrated health and social care day unit (IHSCDU) affected selected outcomes of functional mobility, number of prescribed medications, and physical and psychological well-being. A secondary aim was to examine the utility of the tools to measure these outcomes in this context; the feasibility of the recruitment and retention strategy and the utility of the comparator group.	Quantitative A pre- and post- comparison pilot study with non-randomised intervention and comparator arms.	Participants and outcomes were identified prospectively. Individuals on the intervention arm (*n* = 207; *n* = 27) had to attend the unit at least 1 day a week. Individuals on the comparator arm (*n* = 74; *n* = 25) had to receive at least one visit a week from the community nursing services. November 2010 and September 2012	The modified Barthel Scale SF-12 Blood pressure BMI No. of medications prescribed	Functional mobility: no significant changes. Physical wellbeing - small improvement in the intervention arm but the comparators declined. Psychological well-being: no significant changes. The number of medications prescribed increased in both arms.
Newall, Menec (53), 2015, Canada, 2015 Targeting Socially Isolated Older Adults: A Process Evaluation of the Senior Centre Without Walls Social and Educational Programme	To examine whether Senior Centres Without Walls (SCWOW) was reaching its target population and to gather participant feedback about programme implementation and the perceived satisfaction and impact of the programme.	Qualitative	Telephone interviews were conducted with 26 participants (92% females; aged 57–85 years). May to December 2011.	Telephone interviews with open-ended questions about access/barrier, satisfaction with the programme and impact.	Participants reported having no difficulty using the telephone system. They were very satisfied with the programme and reported that SCWOW have positive effects, e.g., connecting to the larger community, affecting mental well-being. Importantly, no barriers to participation were identified.
Pitkala, et al. (40), 2011, Finland	To determine the effects of socially stimulating group intervention on cognition among older individuals suffering from loneliness.	Quantitative A randomised controlled trial.	Two hundred thirty-five participants (≥75 years) in 7 day care centres in Finland. The three-month group intervention utilised closed-group dynamics and peer support to promote social interaction and friendships. Led by two trained professionals, the sessions combined discussions with activities such as therapeutic writing, group exercise, and art experiences tailored to participants’ interests.	Cognition was measured by the Alzheimer’s Disease Assessment Scale (ADAS–Cog), and mental function was measured by the 15D measure.	At baseline, the intervention and control groups were similar. After 3 months, cognitive function (ADAS–Cog) improved more in the intervention group, and at 12 months, mental function (15D) was significantly better. Overall, the psychosocial group intervention enhanced cognition among lonely older adults
Rowe et al. (33), 2011, USA, Characteristics of Creative Expression Activities: The Links Between Creativity, Failure-Free, and Group Process With Levels of Staff-Participant Engagement and Participant Affect in an Adult Day Centre (ADC)	To assess the relationship between three characteristics identified as central to creative expression (CE) activities: creativity, failure-free, and group process with staff-participant engagement and participant affect.	Quantitative An observational study with a time-sampling design of 10-min periods	Participants included 32 ADC clients and 19 staff members who were engaged in structured ADC activities. All ADC participants were community dwelling. Ages 55–90 years (m = 78.25). Most observed clients were female (74%) with mild to moderate dementia (71%). The majority of staff members were certified nursing assistants (53%), social workers (11%), and were activity staff (37%). All staff members were female and had been employed at the centre an average of 8.5 years.	Staff interactions were observed 1–3 h on 4 separate days a week during a period of 8 weeks. Each 1-h observation was divided into 4 10-min periods. Presence or absence of three traits: creativity, failure-free, and group process to measure: Number of eye-contact Interest in others’ work Talking with a participant on their level Touch: demonstrating care or support Verbal, or conversations about good things Philadelphia Geriatric Centre Affect Rating Scale	Greater levels of positive effect were observed when participants were involved in activities that included creativity or group process.
Sarkar et al. (41), 2017, India, Impact of attendance in a daycare centre on depression among elderly in rural Puducherry: A pre- & post-intervention study	To assess the impact of attendance at a community-based daycare centre in rural Puducherry, India, on depression, cognitive impairment (CI) and QOL of the elderly.	Quantitative Pre- and post- intervention study design	Participants who were allocated and received the intervention at a day care centre (*n* = 263) and after (*n* = 242). January 2013 to January 2014	Geriatric Depression Scale (GDS, short form) Mini-Mental Status Examination (MMSE) World Health Organisation measuring Quality of Life - shortened version (WHOQOL-BREF)	Attendance at the daycare centre reduced the probability of depression by about 51 per cent. An improvement in the WHO QOL scores in the social domain among those who attended more than once a month.
Schmitt et al. (42), 2010, USA, Adult day health centre participation and health-related quality of life	To assess the association between Adult Day Health Centre (ADHC) participation and health-related quality of life.	Quantitative A Case-controlled prospective study design	Comparison of a convenience sample of newly enrolled participants (*n* = 143) from 16 ADHC programmes in six counties of the San Francisco Bay Area with community-dwelling older adults (*n* = 127) from the same geographical area, who did not attend an ADHC. January 2001 to April 2004	SF 36 Physical Self-Maintenance Scale (PSMS) MMSE GDS Charlson Comorbidity Index (CCI)	One year after enrolment, the SF-36 domains role physical and role emotional improved significantly. Adjusted role physical scores for ADHC participants improved (23 vs. 36) but declined for the comparison group (38 vs. 26, time by group interaction *p* = 0.01), and role emotional scores improved for ADHC participants and role emotional scores for ADHC participants.
Shoesmith et al. (34), UK, 2021 Acceptability and feasibility study of a six-week person-centred, therapeutic visual art intervention for people with dementia	To address issues of feasibility in delivering a newly developed, person – centred therapeutic visual art intervention.	Quantitative A mixed-methods, quasi-experimental, pre/post design	Person-centred visual art intervention for people with dementia.	Semi-structured interviews	Five themes were identified from the interviews. Two themes reflected the feasibility/acceptability and the perceived impacts of the intervention, and three themes represented perceived successful elements: participant choice, socialisation and mentally stimulating activities. The quantitative data tentatively indicated enhanced social functioning and quality of life scores post-intervention. The findings indicate that engagement with visual art is effective for people with dementia, and taking into account
Straubmeier et al. (35), 2017, Germany, Non-Pharmacological Treatment in People With Cognitive Impairment	To test the following research hypothesis: MAKS therapy in day care leads to significantly better progression of activities of daily living (ADL) abilities and cognitive abilities of individuals with mild cognitive impairment (MCI) or dementia than treatment as usual in the control group.	Quantitative A cluster-randomised, controlled, multicentre, prospective study.	The sample consisted of 362 individuals, excluding the 91 dropouts. Sixty-one percent of the day care centre users in the sample were women, and users’ mean age was 81.3 years (standard deviation [SD] = 7.5). 1 April 2015 and 31 March 2017, 6-month intervention phase	MMSE, Montreal Cognitive Assessment (MoCA) Erlangen test of activities of daily living (ETAM)	The intervention group (MAKS) had significantly better MMSE and ETAM scores than the control group.
Tretteteig (36), 2017, Norway, 2017 The influence of day care centres designed for people with dementia on family caregivers - a qualitative study	To provide an extended understanding of the situation of family caregivers and to examine to what extent day care centres can meet their need for support and respite.	Qualitative Descriptive design, Text condensation.	The study consisted 17 caregivers of persons with dementia attending day care centres. March and April 2015	In-depth interviews	Positive changes in the relationship. Relieves family caregivers by meeting the person with dementia’s needs. A higher quality of time spent together and easier cooperation, but it also produced some hard feelings and challenging situations.
Weintruab-Youdkes et al. (37) 2015, Israel, A Novel Modification of the “Method of Loci” to Improve Memory in Older Adults	To examine if modified “Method of Loci” method improves memory function.	Quantitative A pilot study	The sample consisted of 22 participants (Female *n* = 19 and male *n* = 3). The mean age was 80.6 ± 6.4 years; the mean years of education was 7.1 ± 5. Mean MMSE before training was 26.4 ± 2.8, and MMSE score did not change following the training.	Computerised neurocognitive tests with regular attends to an adult day care centre	Significant improvement over time in global cognitive function and in memory function.

## Results

The result are presented in text and in greater detail in Table 1, which is structured based on the five research questions. A total of 29 articles (quantitative *n* = 23, qualitative *n* = 6) were included (Table 1). The studies were conducted in countries and region: Canada (*n* = 1), Finland (*n* = 1), Germany (*n* = 1), India (*n* = 1), Iran (*n* = 1), Israel (*n* = 5), Italy (*n* = 1), Japan (*n* = 1), Netherlands (*n* = 1), Norway (*n* = 5), Spain (*n* = 2), Taiwan (*n* = 2), United Kingdom (*n* = 2) and United States (*n* = 4; Table 1).

### Interventions at senior day centres

The most common interventions at senior day centres were various types of group activities with a social focus, such as music, choirs and bands, art and crafts, cooking, playing board games and gardening (15, 25–37). Some interventions involved interactions with the older adults, such as reading newspapers, conversation in groups, playing theatre, and language and computer classes (15, 25, 31, 34, 35, 37–42). There were also interventions based on specific dates in the calendar such as Easter and Valentine’s Day, as well and activities related to local history and culture, including tours to local sites of historical interest (32, 43). The interventions included activities targeting physical, cognitive and social functioning (26, 28, 34, 35, 37, 40, 43–48). For example, interventions focused on social functioning might involve health education, medical check-ups or nursing services (42, 46), but also social activities such as taking coffee breaks and meals together during the day (35, 42, 49). There were some interventions involving problem-solving therapy (intended to improve communication skills) (38, 41), humour therapy (50), activities designed specifically for people with dementia (25, 34–36, 45, 48, 51, 52) and for socially isolated older adults (40, 44, 53). Physical interventions included physiotherapy sessions such as mobility games focusing on strength and balance training (29, 32, 35, 37, 44, 47–49), dance, exercise, tai chi, table tennis, yoga, aerobics, gateball, and general mobility exercises to improve gross and fine motor skills. Interventions related to cognitive function were performed by occupational therapists and could include cognitive activation, memorising, recognition, forming associations, special cognitive abilities such as language comprehension, and logic exercises using pen-and-paper (35, 41), sometimes combined with training in the activities of daily living (ADL) (41). Activities such as beauty treatments and bathing and services such as laundry and transportation were also offered, usually characterised as welfare-related (49).

### The outcome dimensions used to capture the effects of the intervention

The outcomes following the intervention at senior day centres were categorised in domains and elaborated upon in the text and partially described in Table 2. The results from the interventions demonstrated improvements for example in domains as physical ability, cognitive ability, social relationships and ADL. The positive outcome dimensions were mainly improved physical ability (26, 29, 32, 42–44, 46–49), followed by improved cognitive ability (26, 29, 35, 37, 41–43, 47), health-related quality of life (28–30, 32, 41–43, 49), general wellbeing (25, 30, 32, 44, 46, 50, 53) and social relationships (28, 30, 38, 39, 51). User satisfaction (33, 36, 38, 52), ADL (26, 30, 32, 35, 42, 47), depression (41, 42, 46), comorbidity (42), loneliness (28, 39, 40, 44) and self-esteem (28) were other outcome dimensions described in the studies. The outcome dimensions for user satisfaction had increased and included the participants’ communication and interactions with staff (33, 38, 52).

**Table 2:** Overview of main findings of the interventions at senior day centres for older adults (category/area, type of intervention occurrence and reference number).

Category/(Area)	Type of intervention	Occurrence *n* = 29	Reference number
Physical ability	Multifactorial intervention, Fall prevention, physical training sessions, music therapy, farm-based therapy and VR fitness games	11 (38%)	(25, 28, 31, 34, 41–47)
Cognitive ability	Computerised tests, Multifactorial intervention and/or physical training, VR fitness games, Music therapy	8 (28%)	(25, 28, 34, 36, 40, 42, 43, 46)
Social ability	Multifactorial intervention, Art, Sing (for health), Knitting, Humour	8 (28%)	(26, 27, 29, 34, 39, 43, 50, 51)
Activities of daily living (ADL)	General participating in senior day care centre services Multifactorial intervention	5 (17%)	(25, 29, 34, 41, 46)

### Tools/metrics used in interventions at senior day centres

Various tools and metrics were used in the studies. These can be divided into four different areas: *health-related quality of life*, *user satisfaction, cognitive ability and ability to perform activities of daily life (ADLs)*, and *health conditions.* Data from three of the qualitive studies (25, 34, 36) describe the participants’ perceptions of the interventions and were related to general wellbeing in the area of *user satisfaction*.

#### Health-related quality of life

Seven studies (29, 30, 32, 41–43, 49) used various outcome measures to describe quality of life and six studies used assessments of general wellbeing (27, 28, 32, 46, 50, 53). The measurements used in these studies were: the Short Form Health Survey 36 (SF 36) (54), Self-Rated Health, Korean version of Short-form 36 (55), the Short Form Health Survey 12 (56), the World Health Organisation Quality of Life (WHO QOL) (57) and The World Health Organisation Quality of Life-OLD (58). To describe general wellbeing measurements, tools such as the RAND Health Status Questionnaire shortened version (59), Satisfaction with Life Scale (60), Scale of Social Well-Being (61), MacArthur Scale of Subjective Social Status (62), Rosenberg Self-Esteem Scale (63), General Well-Being Schedule (GWB) (64), The Oslo Social support Scale (OSS-3) (65) and Brief Symptom Inventory (BSI) (66) were used.

#### User satisfaction

User satisfaction was observed, measured and evaluated in five studies (27, 33, 36, 38, 52). Two studies used a self-assessment questionnaire (38, 52) and one in-depth interviews (36). In the study by Rowe et al. (33) and Eaton (27), the interactions between staff and older adults were observed and measured when performing various group activities. User satisfaction was also assessed on the Philadelphia Geriatric Centre Affect Rating Scale (ARS) (67).

#### Cognitive ability and ability to perform ADLs

Ten studies (26, 29, 35, 37, 40–43, 45, 47) measured changes in cognitive ability related to an intervention. Four studies (35, 37, 41, 42) used the Mini Mental Status Examination (MMSE) (68) to assess cognitive function. The Abbreviated Mental Test Score (69), Mental Component Summary Score in SF-36 (54), Montreal Cognitive Assessment (MoCA) (70), Short Portable Mental Status Questionnaire (SPMSQ) (71), The Alzheimer’s Disease Assessment Scale (ADAS-Cog) (72), Clinical Dementia Rating (CDR) Scale (73), Executive Interview (EXIT-25) (74), Chinese Version of the Verbal Learning Test (CVVLT) (75) and near-infra red spectroscopy (NIRS) were also used.

ADLs were described and measured in six of the included studies (26, 30, 32, 35, 42, 47). Tools used in these studies included the Katz Index of Independence in Activities of Daily Living (76), the Erlangen Test of Activities of Daily Living (ETAM) (77) and the modified Barthel Index (78). Fillenbaum’s (78) and Lawton’s (79) instruments were also used to examine the ability to perform instrumental activities of daily living (IADLs).

#### Health conditions

All of the studies described various health factors such as body weight, height, socioeconomic status, blood pressure and chronic conditions without any specific measurements. Some specific metrics of physical aspects such as walking (80), balance (81), muscle power (82), handgrip strength (83), curvature of the spine (using Spinal Mouse®) (84), and sleep (using ActiSleep) (85) were assessed in the studies (26, 42, 43, 45). The Physical Self-Maintenance Scale (PSMS) (79) was also used. Sarkar et al. (41) and Schmitt et al. (42) used the Geriatric Depression Scale (GDS) (86) to measure depression. Loneliness was measured on the UCLA Loneliness Scale (87) in the studies by Ayalon et al. (39) and Galinha et al. (28) respectively with Beck Depression Inventory (BDI) (88) in the study by Böen et al. (44). Risk of falls was measured using the Tinetti test (89) and the Short Physical Performance Battery (SPPB) (90). One study (42) presented comorbidity among the older adults using the Charlson Comorbidity Index (CCI) (91).

### The effects of interventions or programmes

Participating and having multifactorial interventions (26, 29, 30, 32, 35, 41–43, 45–47, 49) at senior day care centres including physical, cognitive and psychosocial aspects seemed to be of importance in order to increase or maintain abilities in older adults. Overall, the most common effect of interventions was on quality of life (28–30, 32, 41–43, 49) and wellbeing (32, 40, 46, 50, 53). Physical improvements in older adults were for example found in interventions involving fall prevention, physical training sessions, music therapy, farm-based therapy and VR fitness games (26, 43, 45–49). Improvements in cognitive ability and the ability to perform activities of daily living between groups were also seen in some studies (35, 47) as well as gradual improvements in global cognitive function and memory function when using computerised neurocognitive tests (37). Another positive effect observed was that attending senior day centres could reduce the probability of depression by about 51%, There were also improvements in the WHO QOL scores in the social domain among those attending centres more than once a month (41). Other benefits of attending senior day centres included reduced loneliness and improved sociability (28–31, 39, 40, 44, 51). Participating in creative group interventions had a positive impact on social interactions and relationships in older adults with or without dementia (26, 28–31, 33, 51). The use of humour- (50), art- (27), respectively singing- (28) therapy was associated with positive effects on mental health, including lower levels of anxiety and depression, as well as improved general wellbeing but should be examined in further studies (50) (Table 2).

### Professions involved in the interventions

The interventions included a broad range of professionals and contributors with varying roles and qualifications. Professions involved in interventions included healthcare professionals such as nurses, assistant nurses, occupational therapists and physiotherapists, as well as social workers and activity staff (25, 26, 31–33, 35, 36, 38, 41, 42, 45–53). OTs or PTs often led tailored group activity sessions, and physical sessions. Others involved were exercise specialists, healthy volunteers, a humourist, art and music teachers, visual art facilitators, family caregivers and other relatives (26, 27, 29, 34, 37, 43, 50, 53). These were often responsible for promoting general well-being, psychosocial support, and fostering social interaction.

## Discussion

The findings of this scoping review indicate substantial variation in the types of interventions, outcome measures, reported effects, and professional involvement, reflecting the complexity of the field. The studies we have examined also represent a range of settings in which senior day centres deliver a variety of programmes. Attending a senior day centre can be valuable for older adults, helping them to maintain or improve their physical, cognitive and social functioning. However, this scoping review reveals the difficulty in describing the most effective, evidence-based rehabilitation interventions for older adults attending senior day centres. This highlights the need for rigorous research to identify the most effective intervention components and their impact on key outcomes among older adults attending senior day centres.

Older adults participating in activities at senior day centres can be supported and engaged in various interventions, including social interactions or activities that can improve or maintain their ability to age in place (2, 4, 92). Our results are in line with previous research in as much as the interventions implemented at the senior day centres often involved group activities with a social focus, such as music, crafts and board games (26–28, 31, 33, 34, 50), and therapeutic activities intended to improve social, physical, and/or cognitive functioning (25, 29, 30, 35, 37, 38, 43, 45–48, 51) and illustrated in Tables 1, 2. These various activities are of importance to supply with regular physical activity and these activities may also be person-centred, meaning they can be tailored to what is meaningful and important in the older adult’s daily life, supporting for example maintain abilities to age in place and quality of life (11, 43, 92).

One question that does arise is whether and if so to what extent older adults have the opportunity to choose an activity when attending a senior day centre. Studies have shown that meaningful participation is often dependent on identifying individual wishes and needs, thus facilitating person-centred interventions (16, 92). In their study, Brataas et al. (25) touch on this, noting that the occupational therapist organising the programme at one senior day centre did so together with the participants. However, it is unclear how the participants’ individual wishes and needs were assessed. If individual needs are not met, this may mean that older adults participating in activities at social day centres are less motivated, despite the fact that they would probably ‘benefit from it’. Moreover, the group of older adults can hardly be viewed as homogeneous, meaning that rehabilitation interventions at senior day centres should be based on person-centred plans and activities. Senior day centres also appear to have weaknesses when it comes to implementing preventive interventions to promote healthy ageing; developing health-promotion strategies to support functional ability should be of importance and may promote healthier ageing among the older adults who attend senior day centres (93). To ensure evidence-based practice at senior day centres, findings from related settings, such as long-term care facilities and nursing homes could be tested and integrated to a higher degree. As these studies have shown, i.e., multicomponent exercise programme improved general functionality as strength, balance, blood pressure and fall risk in older adults (43, 94, 95). This approach can help validate interventions and support adaptation in the senior day centre context, ultimately leading to more effective and sustainable health-promotion strategies.

The outcome dimensions use in the studies covered in this scoping review were mainly focused on physical and cognitive ability (26, 29, 30, 35, 37, 41–43, 45, 47), health-related quality of life (28, 30, 42, 49, 50) and/or social relationships (28, 39, 51). This is hardly surprising, as experienced health problems, support needs and cognitive ability change with age (96). Measuring cognitive ability is also important given that maintaining cognitive ability is one of the main determinants of whether an individual can continue to live independently. However, it was surprising that structured fall risk assessments are not conducted more extensively. Falls are a leading cause of injury, hospitalisation, and loss of independence among older adults (97). Therefore, implementing systematic fall risk assessments should be considered as a component of preventive care at senior day centres, helping to identify at-risk individuals and guide interventions to maintain safety and functional ability (95, 97). It would therefore be extremely valuable to examine these aspects in a structured manner at senior day centres. Regarding the activities of daily living, this was often measured on the Barthel Index (26, 32, 48) or some other measure of IADL (30, 35, 47). So, to a large extent the effects of interventions in senior day centres focus on ability to perform ADLs. This is an important reflection, since rehabilitative interventions at senior day centres not only strive to maintain independence and quality of life, but also to promote general ability and social engagement with other people. It is therefore crucial to take activities other than ADL into account as outcome dimensions, such as social and leisure activities. Previous research has found that the ability to maintain social relationships or social activities is important to maintaining everyday activities at home (5, 98). This aspect has been described by Lees (99), who questions how various professionals were able to identify older adults with a fragile social support network living in involuntary loneliness at home. On the other hand, there might be cultural differences between the countries represented in the studies included in this scoping review. Previous research (100, 101), has indicated that senior day centres can function as arenas for interpretation and translation, thereby fostering enhanced wellbeing among older adults. Furthermore, staff members were found to play a crucial role in facilitating connections between older adults and other key stakeholders within the care system, such as the municipalities and additional service providers. These findings are consistent with and further supported by the results of the present scoping review. Greater attention must therefore be paid to outcome dimensions that measure social support or social network along with functional needs at senior day centres.

Overall, interventions at senior day centres had a greater impact on quality of life and wellbeing than on loneliness and social status. One important effect of participating in interventions at senior day centres was that older adults showed higher levels of subjective social status and lower levels of loneliness (39). Since many older adults experience loneliness and desire social inclusion (13), it is important to reach those in this group of older adults and offer individualised interventions at senior day centres. A key solution for participating in interventions at senior day centres may be to participate for only part of the day or having regular telephone contacts as described in the study by Newall et al. (53).

A variety of professional disciplines were represented across the studies, highlighting the diverse expertise engaged in delivering the interventions. The professions involved in the studies were often nurses, assistant nurses, OTs, and PTs, who provided rehabilitation including for example assessments and various group activities. Furthermore, there are some unclear designations of staff working at the senior day centres, such as medical professionals and humourist. “Healthy volunteers” were involved in the study by Battaglia (43), but it is not clear what this means. This leads to a lack of clarity regarding the profession’s role and responsibility in performing the interventions at the senior day centres. Moreover, it is unclear which professions are most suited to the role. This diversity reflects the interdisciplinary nature of the interventions and the multifaceted approach to promoting health and well-being in senior day centres. There are some important questions to be answered about staffing at senior day centres, including whether licensed healthcare professionals are required and who has the requisite knowledge or is best suited to assessing individual needs, offering meaningful activities and facilitating participation. Multidisciplinary teams are essential in geriatric rehabilitation. Effective collaboration among professionals can lead to better care coordination and supports healthy ageing (102, 103) where professionals as assistant nurses (for engagement and participation in meaningful activities), OTs (for cognitive and ADL ability), PTs (for physical ability) and social workers (for emotional and service support) should be the minimum requirements for core competencies at senior day centres. In addition, the reviews by Fields et al. (104) and Lunt et al. (92) describe a lack of standardised definitions of services and content at senior day centres, something that makes any assessment and evaluation of efficacy difficult. In conclusion, to strengthen the evidence base and inform clinical practice, future research should include large-scale, methodologically robust randomised controlled trials incorporating results from related contexts as long-term care facilities and nursing homes (43, 94, 95). Such studies would also benefit from incorporating structured multidisciplinary person-centred rehabilitation interventions. The interventions should be guided by the principles of person-centred care, meaning that the individual’s goals, preferences, values, and lived experiences are central to the planning and delivery of care (105, 106). In addition, the interventions should be supported by MRC Guidance (107) to leverage technological solutions, ensuring active stakeholder involvement, and combining quantitative and qualitative methodologies to capture both outcomes and contextual factors.

### Strength and limitations

Using the two databases CINAHL and PubMed might be a limitation and searching other databases such as the Cochrane Library, and PsycInfo may have yielded other published papers relevant to the aims of this scoping review. The authors involved in this study have prior experience of working with older adults and various rehabilitative interventions. Previous experience can both support and limit the research in terms of formulating research questions and data analysis. To enhance the credibility in this study, continuous engagement with data were performed by first and last author and continuously discussed by all authors. This process is a form of triangulation, which is considered to strengthen trustworthiness of the findings (108). A key strength of this scoping review lies in its comprehensive overview of interventions in senior day centres for older adults, including how these have been reported and categorised. Even if the interventions and outcome dimensions varied, the findings are an important first step towards developing complex interventions for older adults attending senior day centres (109). This scoping review has also highlighted the need for an appropriate framework to specify and describe the interventions at senior day centres for older adults. On the other hand, factors such as culture, relative income, and subjective well-being may vary among individuals in different countries or regions. Nevertheless, individuals with higher relative income, as well as those who, for instance, accept income inequality, have been found to report higher levels of subjective well-being (110) which is in line with the results in this study. A framework defining the concept of senior day centres may help those involved to tailor or choose interventions that promote a social and active life for older adults.

## Conclusion

This scoping review revealed that, despite diverse interventions delivered in senior day centres, evidence remains insufficient to determine which interventions are the most effective. Greater focus on person-centred approaches and rigorous evaluation of intervention components, outcomes, and professional roles is needed to strengthen the evidence base for rehabilitation in this setting.

### Implications for rehabilitation

- Senior day centres for older adults are poorly organised and described to address person-centred needs in older adults.
- Future initiatives should prioritise the development of structured assessment procedures and targeted interventions at senior day centres to enhance their role in supporting ageing in place.
- Clear handovers and documentation of rehabilitation outcomes interventions from senior day centres for older adults to outpatient healthcare are essential.
- Adequate levels of rehabilitation competence must be ensured among staff at senior day centres.

## Data Availability

The original contributions presented in the study are included in the article/supplementary material, further inquiries can be directed to the corresponding author.
